# Cardiac and vascular autonomic control in patients with hereditary angioedema

**DOI:** 10.3389/fphys.2025.1690915

**Published:** 2025-12-16

**Authors:** Beatrice De Maria, Luca Ranucci, Clara Gino, Aida Zulueta, Monica Parati, Azzurra Cesoni Marcelli, Lorenza Chiara Zingale, Riccardo Sideri, Laura Adelaide Dalla Vecchia, Francesca Perego

**Affiliations:** 1 Istituti Clinici Scientifici Maugeri IRCCS, Bioengineering Laboratory, Milan, Italy; 2 Istituti Clinici Scientifici Maugeri IRCCS, Department of Internal Medicine and Rehabilitation, Milan, Italy; 3 Istituti Clinici Scientifici Maugeri IRCCS, LaBioN, Milan, Italy; 4 Istituti Clinici Scientifici Maugeri IRCCS, Department of Cardiology, Milan, Italy

**Keywords:** hereditary angioedema, rare disease, long-term prophylaxis, heart rate variability, systolic arterial pressure variability, autonomic nervous system, secondary prevention

## Abstract

**Background:**

Regulation of vascular permeability in hereditary angioedema due to C1 inhibitor deficiency (HAE-C1INH) is key to understanding the disease, but the role of the autonomic nervous system (ANS) in this mechanism remains unclear.

**Purpose:**

The aim of this study was to compare the cardiovascular autonomic response to the head-up tilt test (HUTT) in HAE-C1INH patients and matched healthy controls (HCs).

**Methods:**

HAE-C1INH patients were evaluated during a 1-week symptom-free period. Electrocardiogram (ECG) and beat-to-beat non-invasive arterial blood pressure (BP) were recorded in the supine position (REST) and during 70° tilt (TILT). Heart rate and systolic BP (SBP) variability indices were derived. Variance (σ^2^_SBP_) and low-frequency power (LF_SBP_) of SBP variability were used as markers of sympathetic vascular control.

**Results:**

Twenty-five HAE-C1INH patients [13 male individuals, 44 (28.8–57.5) years] and 25 HCs [13 male individuals, 44 (30.8–54.3) years] were enrolled and divided into <45 and ≥45 age groups. Eighteen patients were on long-term prophylaxis (LTP). In the younger group, LF_SBP_ increased from REST to TILT in both groups, with no differences. In older subjects, HAE-C1INH patients showed higher σ^2^_SBP_ [21.2 (9.3–59.2) vs. 7.5 (1.9–14.6) mmHg^2^] and a greater LF_SBP_ increase [9.4 (4.6–22.4) vs. 0.9 (0.2–7.6) mmHg^2^] than HCs during TILT, suggesting sympathetic hyperactivation. No significant group differences in cardiac autonomic control were observed during REST or TILT, regardless of age. Findings in the LTP subgroup mirrored those of the full cohort.

**Conclusion:**

Older HAE-C1INH patients display altered vascular autonomic regulation, with an exaggerated sympathetic response during orthostatic stress. Further studies are needed to assess the role of LTP in these alterations.

**Clinical Trial Registration:**

https://clinicaltrials.gov/study/NCT06408805?cond=Hereditary%20Angioedema&term=autonomic&rank=1

## Background

Hereditary angioedema due to C1 inhibitor deficiency (HAE-C1INH type 1 and type 2) (Reshef et al., 2024) is a rare disease with a prevalence of 1.22 cases per 100,000 people (Fisch et al., 2025). It is characterized by a localized, self-limiting subcutaneous or submucosal edema associated with different mechanisms (Depetri et al., 2019) that control endothelial barrier function (Reshef et al., 2024). The symptoms encompass swelling of the extremities, genitals, bowel mucosa, face, and the upper airway, including the larynx. Laryngeal attacks, if not treated, can lead to death (Busse and Christiansen, 2020). HAE attacks are characterized by their unpredictability and episodic nature, arising from the release of the principal mediator, namely, bradykinin (BK), consequent to hyperactivation of the contact system lacking its primary regulatory protein (C1-INH) (Cicardi et al., 1998). The resultant impairment of endothelial function is accompanied by an increase in vascular permeability, which in turn allows local and unpredictable release of BK.

The autonomic nervous system (ANS) plays a key role in vascular permeability regulation (Marshall, 1982), an essential process in the pathophysiology of HAE-C1INH. Experimental studies suggest that the sympathetic nervous system influences microvascular permeability, as in the case of the α2 agonist clonidine, which reduces capillary leakage in inflammatory states (Schmidt et al., 2015). These findings raise the question of whether autonomic dysfunction contributes to the unpredictable nature of angioedema attacks and whether targeting the ANS could offer novel therapeutic strategies. In addition, the protective role of the vagus nerve in models of inflammation, such as ischemia–reperfusion injury, has been well-documented (Jiang et al., 2014; 2022). The parasympathetic tone, acting on B2 receptors in the nucleus ambiguus, can also be modulated by BK (Brailoiu et al., 2017).

In current clinical practice, investigation of ANS activity directed at the cardiovascular system is typically undertaken using the head-up tilt test (HUTT) (Brignole, 2018). Power spectral analysis of heart rate variability (HRV) and arterial blood pressure variability (BP) assesses the spontaneous fluctuations of heart period and vessels modulation. This analysis of data collected during HUTT derives indices of the cardiovascular autonomic control (Cheshire et al., 2021).

Previous studies have investigated the cardiac autonomic regulation in HAE-C1INH patients across different disease phases, including prodromal states, active angioedema attacks, and remission (Perego et al., 2021). Findings suggest that HRV analysis, particularly when extended to multiday electrocardiogram (ECG) recordings, may serve as an early marker of impending attacks (Perego et al., 2021). This supports the hypothesis that autonomic imbalance plays a role in the onset of symptoms (Wu et al., 2020). However, the vascular autonomic function during remission periods has been investigated in basal conditions and during HUTT in a single study (Wu et al., 2017). Unfortunately, more than 50% of patients on long-term prophylaxis (LTP) with attenuated androgens were included in the investigation. This medication is known to interfere with the ANS function. Androgens enhance norepinephrine release and heighten receptor sensitivity, and the reduction of estrogen levels due to danazol therapy may lead to decreased vagal (parasympathetic) tone, which could contribute to an autonomic imbalance favoring sympathetic dominance. In recent years, the availability of new effective prophylactic drugs with different mechanisms of action (Betschel et al., 2023) has completely changed the disease control methods (Beard et al., 2022), but their impact on ANS function is unexplored. Finally, ANS balance changes as age progresses (Accardo et al., 2021; Beard et al., 2022), and the contributions of aging and disease duration in HAE-C1INH have never been considered.

HRV analysis, with its ability to capture subtle fluctuations in autonomic modulation, remains the best method to investigate these potential changes.

## Aim

The aim of the study was to investigate the cardiovascular autonomic control in HAE-C1INH patients compared with that in healthy controls (HCs) during HUTT. Subgroup analyses were performed to consider the contributions of LTP and age.

The primary outcome of the study is the difference in autonomic indices, derived from the time and frequency analysis of the cardiovascular signals, between HAE-C1INH patients and HCs.

## Population and experimental protocol

### Population

The HAE-C1INH patients belonging to ITACA (Italian Network for Hereditary and Acquired Angioedema) were consecutively enrolled at the IRCCS Istituti Clinici Scientifici Maugeri in Milan during an ambulatory visit from July 2023 to March 2024. To assess the primary aim, we calculated the sample size of the study based on the power of the HRV in the high-frequency band, assuming a power of 90%, a significance level of 5%, and an enrollment ratio of 1:1 between controls and patients. The results indicated a required sample of at least 27 subjects per group. The inclusion criteria were age between 18 and 65 years and a documented diagnosis of HAE-C1INH, defined according to guidelines (Maurer et al., 2022). The exclusion criteria were any chronic disease (i.e., hypertension, previous myocardial infarction, diabetes, chronic heart failure, autoimmune disease, and neurodegenerative diseases) or medication influencing the ANS (beta-blockers, calcium channel blockers, tricyclic antidepressants, and sympathomimetics), active acute diseases, experiencing syncope during HUTT, and an acute attack experienced within the previous week and within 72 h after the experimental protocol (*a posteriori* exclusion). Sex- and age-matched HCs were recruited from the workers of the hospital and patients’ relatives. The healthy status of HCs was assessed by a physician at enrollment. SARS-CoV-2 infection within the preceding 3 months and a history of syncope (Porta et al., 2023) were reasons for exclusion in both patients and controls.

### Experimental protocol

On the day of enrollment, the patients and HCs signed their written informed consent. A physician collected the demographic and clinical characteristics and scheduled the HUTT.

The HUTT was performed in the morning (between 9 and 12 a.m.), after a well-slept night, in a quiet room with comfortable temperature. Participants abstained from caffeinated or alcoholic beverages for 12 h and from strenuous physical activity for 72 h prior to the test (Dalla Vecchia et al., 2014). The HUTT consists of monitoring the ECG, respiration, and non-invasive BP for 10 min in the supine position (REST) and for 10 min during passive orthostatic position at 70° (TILT). ECG was obtained from a modified lead II via the LAB3 device (Marazza, Monza, Italy) and sampled at 1,000 Hz. BP was recorded using a photoplethysmography device (Finometer, Finapres Medical Systems) at the middle finger of the right hand and sampled at 250 Hz. Thoracic excursion was recorded via a strain-gauge belt positioned on the subjects’ thorax. Patients were instructed not to speak during the test and to breathe spontaneously. In case of the occurrence of presyncope signs during the tilting phase, the supine position was immediately restored.

The experimental procedures were performed according to the Declaration of Helsinki, and the protocol was approved by the Istituti Clinici Scientifici Maugeri Ethics Committee on 21 February 2023 (approval number 2746). The registration number at ClinicalTrial.gov is NCT06408805.

## Methods

### Signals pre-processing

The signal pre-processing consisted of the beat-to-beat variability series extraction from the ECG and BP signals. From the ECG, the RR interval (RR) was derived as the temporal distance between two consecutive R peaks detected on the ECG. The QRS complex was detected using an automatic algorithm based on a threshold applied to the first derivative of the signal, with the R peak determined using parabolic interpolation (Porta et al., 1998). The beat-to-beat systolic BP (SBP) series was obtained, identifying the maximum of the BP signal inside each RR.

The obtained series were visually inspected by a trained technician to avoid misdetections. In the case of ectopic beats or artifacts, cubic spline interpolation was performed. The cut-off for acceptable corrections was set at 5%.

For each subject, synchronous segments of 250 consecutive RR and SBP values were identified during REST and TILT conditions for further analysis. Both the REST and TILT segments were identified for all subjects within the 10-min period, excluding the first and last 2 minutes.

### Cardiovascular neural control indices calculation

The characterization of cardiovascular control was performed by calculating the time- and frequency-domain indices for each subject during REST and TILT over the selected segments of RR and SBP time series (Task Force of the European Society of Cardiology and the North American Society of Pacing and Electrophysiology, 1996).

Time-domain indices, calculated after linear detrending of the series, were the mean (µ_RR_ and µ_SBP_) and variance (σ^2^_RR_ and σ^2^_SBP_) of the RR and SBP series, respectively. µ_RR_ was expressed in ms, µ_SBP_ was expressed in mmHg, σ^2^_RR_ was expressed in ms^2^, and σ^2^_SBP_ was expressed in mmHg^2^.

Frequency-domain indices were derived by parametric power spectral analysis. The RR and SBP series were first modeled using an autoregressive model whose order was optimized by the Akaike information criterion (Akaike, 1974). According to the central frequency, each component was labeled as a low-frequency (LF, 0.04 Hz–0.15 Hz) or high-frequency (HF, 0.15 Hz–0.40 Hz) component. The sum of the power spectral HF components of the RR series (HF_RR_) was taken as an index of the cardiac vagal modulation directed to the sinus node (Akselrod et al., 1981). The sum of the LF power spectral components of the SBP series (LF_SBP_) was considered an index of the sympathetic modulation directed to the vessels (Pagani et al., 1986). HF_RR_ was expressed in ms^2^, while LF_SBP_ was expressed in mmHg^2^. The LF component of RR variability was not used as an index of cardiac sympathetic modulation because of the controversial interpretation of this marker (Rahman et al., 2011).

### Statistical analysis

The Shapiro–Wilk test was applied to test the normality of each variable prior to statistical analysis. An unpaired Student’s t-test or Mann–Whitney rank sum test was performed, when appropriate, to compare the demographic and clinical characteristics of the patients and controls. In the case of categorical variables, a χ^2^ test was applied. The same statistical tests were applied to compare the characteristics of the subgroups of patients when subgroup analyses were performed (i.e., patients on LTP vs. patients without LTP and patients younger than 45 years vs. patients older than or equal to 45 years).

The age of 45 years was selected as it represented the most appropriate compromise, allowing the division of the cohort into two equally represented groups, with a cut-off approximately at the midpoint of the age distribution.

Two-way analysis of variance (one-factor repeated measures, Holm–Sidak test for multiple comparisons) was applied to test differences in cardiovascular control indices between the two different experimental conditions (i.e., REST and TILT) and between the two studied populations (i.e., HC and HAE-C1INH patients) in the case of normal distribution. For non-normal distributions, Friedman analysis of variance on ranks (Tukey’s test for multiple comparisons) was applied. Continuous variables were given as median [IQR] values.

Statistical analysis was performed using a commercial statistical software application (SigmaPlot, Systat Software, Chicago, IL, version 11.0). A *p*-value lower than 0.05 was considered significant.

## Results

### Enrolled population

**FIGURE 1:**
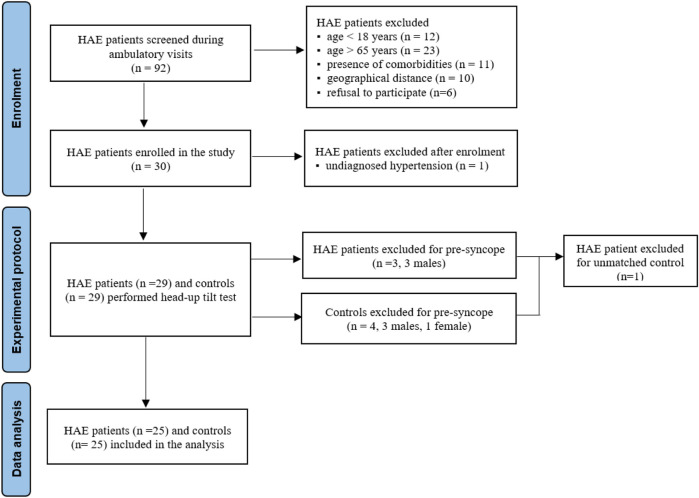
Flowchart of the enrolled population. Legend: HAE, hereditary angioedema.

A schematic representation of the enrolled population is shown in Figure 1. In brief, 92 patients were screened, including 29 HAE-C1INH patients [16 male individuals, median age 44 (27.8–57.5) years] and 29 age- and sex-matched HCs [16 male individuals, age 44 (29.5–54.3) years], who were finally enrolled in the study protocol. In the end, the cardiovascular neural control was assessed in 25 HAE-C1INH patients and 25 matched HCs. The demographic and clinical features of the analyzed population and the related subgroups are presented in Table 1.

**TABLE 1:** Demographic and clinical features of the analyzed enrolled population.

	HC (n = 25)	HAE patients (n = 25)	LTP HAE patients (n = 18)	No LTP HAE patients (n = 7)	HAE patients <45 years (n = 13)	HAE patients ≥45 years (n = 12)
Age, yrs	44 [29.5–54.3]	44 [27.8–57.5]	45.5 [28.0–57.0]	42.0 [29.0–62.0]	28.0 [25.8–37.5]	58.0 [50.0–65.5]$
Sex, males/females	13/12	13/12	10/8	3/4	8/5	5/7
BMI, kg·m^-2^	23.4 [21.1–24.9]	25.0 [23.4–26.0]*	25.0 [24.0–29.0]	23.5 [22.9–25.5]	24.0 [22.7–25.3]	25.5 [24.3–27.5]
Age at HAE diagnosis, yrs	na	19 [8.3–28.3]	18.5 [3.0–28.0]	21.0 [11.5–27.5]	9.0 [3.0–19.5]	26.5 [19.0–33.5]$
Attack rate in the last 6 months	na	0 [0–0.4]	0 [0–0.2]	0.7 [0.1–1.0]#	0.2 [0–1.0]	0 [0–0.1]
No LTP, n (%)	na	7 (28)	0 (0)	7 (100)#	4 (30)	3 (25)
LTP with attenuated androgens, n (%)	na	1 (4)	1 (6)	0 (0)	1 (8)	0 (0)
LTP with lanadelumab, n (%)	na	16 (64)	16 (88)	0 (0)	7 (54)	9 (75)
LTP with C1 esterase inhibitor, n (%)	na	1 (4)	1 (6)	0 (0)	1 (8)	0 (0)
ODT with C1 esterase inhibitor, n (%)	na	4 (16)	4 (22)	0 (0)	4 (30)	0 (0)
ODT with icatibant, n (%)	na	12 (48)	6 (33)	6 (81)	6 (47)	6 (50)
ODT with C1 esterase inhibitor and icatibant, n (%)	na	9 (36)	7 (39)	2 (19)	3 (23)	6 (50)

HC, healthy controls; HAE, hereditary angioedema; BMI, body mass index; LTP, long-term prophylaxis; ODT, on-demand treatment. Numerical values are presented as the median [IQR], while categorical values are presented as the absolute number and percentage in brackets. *, *p* < 0.05 HC vs. HAE patients; #, *p* < 0.05 LTP vs. no LTP HAE patients; $, *p* < 0.05 HAE patients <45 years vs. ≥ 45 years. An unpaired Student’s t-test or Mann–Whitney rank-sum test for continuous variables and a χ2 test for categorical variables were applied.

The results of the comparison between the demographic and clinical features over the whole population of HAE-C1INH patients and HCs revealed that HAE-C1INH patients had a lower body mass index than HCs (*p* = 0.009). Out of 18 HAE-C1INH patients on LTP, 16 were on lanadelumab, while 7 were on on-demand therapy. When the group of patients on LTP (n = 18) was compared to those not on LTP (n = 7), the attack rate calculated over the last 6 months was lower in the former group (*p* = 0.034). Other demographic and clinical characteristics were comparable in the two subgroups. The subgroup analysis of patients younger (n = 13) and equal to or older (n = 12) than 45 years revealed that, in the former group, diagnosis was made earlier than in the latter (*p* = 0.001). The other characteristics were similar in the two subgroups.

### Cardiovascular neural control during HUTT in HAE-C1INH patients and HCs

**FIGURE 2:**
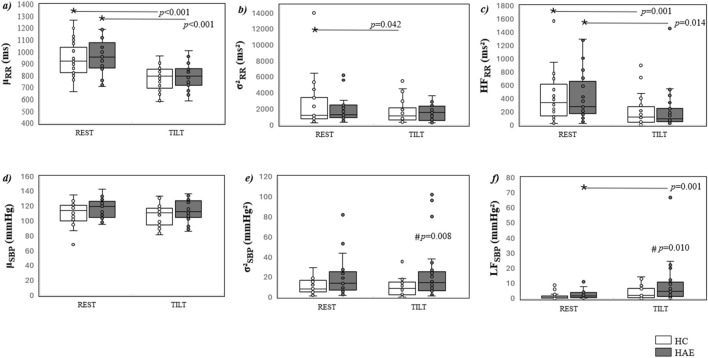
Comparison of the cardiovascular neural control indices in HAE patients and HCs during HUTT. Boxplots show µ_RR_ **(a)**, σ^2^_RR_ **(b)**, HF_RR_ **(c)**, µ_SBP_ **(d)**, σ^2^_SBP_ **(e)**, and LF_SBP_ **(f)** in HAE patients (dark gray boxes) and HCs (white boxes) during REST and TILT. Data are presented as the median [IQR]. *p < 0.05 REST vs. TILT, #*p* < 0.05 HAE patients vs. HCs, based on two-way analysis of variance with Holm–Sidak *post hoc* test.

The results of the comparison between HAE-C1INH patients and HCs in terms of cardiovascular control indices during HUTT are shown in Figure 2. Regarding cardiac neural control, µ_RR_ (Figure 2a) and HF_RR_ (Figure 2c) decreased from REST to TILT in both HAE-C1INH patients and HCs. σ^2^_RR_ (Figure 2b) was unvaried in HAE-C1INH patients from REST to TILT and decreased only in HCs. Regarding the vascular neural control, µ_SBP_ (Figure 2d) did not change between the groups and experimental conditions. σ^2^_SBP_ (Figure 2E) did not vary between the experimental conditions, but it was higher in HAE-C1INH patients than in HCs during TILT. LF_SBP_ (Figure 2f) increased from REST to TILT only in HAE-C1INH patients and was, therefore, higher in HAE-C1INH patients than in HCs during TILT. The *post hoc* power calculation of the results obtained for σ^2^_SBP_ and LF_SBP_ were 70% and 57%, respectively. Even when all the σ^2^_SBP_ values were taken together regardless of the experimental condition (i.e., REST and TILT) and compared, significantly higher values of σ^2^_SBP_ were found in the HAE-C1INH patients’ group than in HCs [14.3 (7.3–24.1) vs. 8.6 (3.9–15.2) mmHg^2^, respectively] (data not shown in the figure).

Respiratory frequency was monitored during both REST [HAE-C1INH, 0.25 (0.21–0.30) Hz; HC, 0.24 (0.20–0.29) Hz] and TILT [HAE-C1INH, 0.24 (0.20–0.29) Hz; HC, 0.23 (0.20–0.32) Hz] conditions and was found to lie within the 0.15 Hz–0.40 Hz range (≈9–24 breaths/min) in all participants. Therefore, the high-frequency component of HRV (HF_RR_) can be reliably interpreted as reflecting vagal modulation.

### Cardiovascular neural control during HUTT in HAE-C1INH patients on LTP and HCs

To verify whether the presence of LTP had an effect on the results described in the previous paragraph, we repeated the same analysis excluding the seven patients who were not on LTP and their matched controls.

The results, summarized in Table 2, are congruent to those obtained in the whole population. The results of the sub-analysis confirmed a decrease in µ_RR_ and HF_RR_ during TILT in both HAE-C1INH patients and HCs, a decrease in σ^2^_RR_ only in HCs, and an increase in LF_SBP_ only in HAE-C1INH patients. σ^2^_SBP_ and LF_SBP_ were significantly higher in HAE-C1INH patients than in HCs during TILT.

**TABLE 2:** Comparison of the cardiovascular neural control indices in the subgroup of HAE-C1INH patients on long-term prophylaxis and their matched HCs during HUTT.

	HC		HC: REST vs. TILT	HAE-C1INH patients on LTP		HAE-C1INH: REST vs. TILT	REST: HAE-C1INH vs. HC	TILT: HAE-C1INH vs. HC
	REST	TILT	*p*	REST	TILT	*p*	*p*	*p*
µ_RR_, ms	940 [847–1,100]	803 [716–864]	<0.001*	980 [883–1,120]	804 [746–856]	<0.001*	0.982	0.668
σ^2^_RR_, ms^2^	2,480 [1,040–3,520]	1,040 [614–2,990]	0.027*	1,270 [982–2,530]	1,310 [455–2,440]	0.456	0.079	0.655
HF_RR_, ms^2^	371 [140–1,230]	121 [27–301]	<0.001*	264 [167–586]	79 [49–226]	0.016*	0.186	0.652
µ_SBP_, mmHg	112.5 [99.0–121.0]	109.5 [93.2–115.0]	0.339	122.5 [104.0–127.0]	114.5 [106.0–127.0]	0.360	0.143	0.133
σ^2^_SBP_, mmHg^2^	10.7 [5.13–18.8]	7.8 [2.3–14.0]	0.928	11.0 [6.1–20.9]	10.8 [6.9–24.1]	0.345	0.093	0.007*
LF_SBP_, mmHg^2^	0.99 [0.51–1.55]	1.3 [0.3–3.7]	0.311	1.7 [0.9–3.8]	4.2 [1.5–6.7]	0.002*	0.671	0.009*

REST, resting position; TILT, head-up tilt test at 70°; HCs, healthy controls; RR, RR interval; µ_RR_, mean of RR series; σ^2^_RR_, variance of RR series; HF, high frequency; HF_RR_, power in the σ band of the RR series; SBP, systolic arterial blood pressure; µ_SBP_, mean of SBP series; σ^2^_SBP_, variance of SBP series; LF, low frequency; LF_SBP_, power in the LF band of SBP series. Data are presented as the median [IQR]. *p < 0.05 based on two-way analysis of variance with Holm–Sidak *post hoc* test.

### Cardiovascular neural control during HUTT in HAE-C1INH patients and HCs: comparison between younger and older groups

**FIGURE 3:**
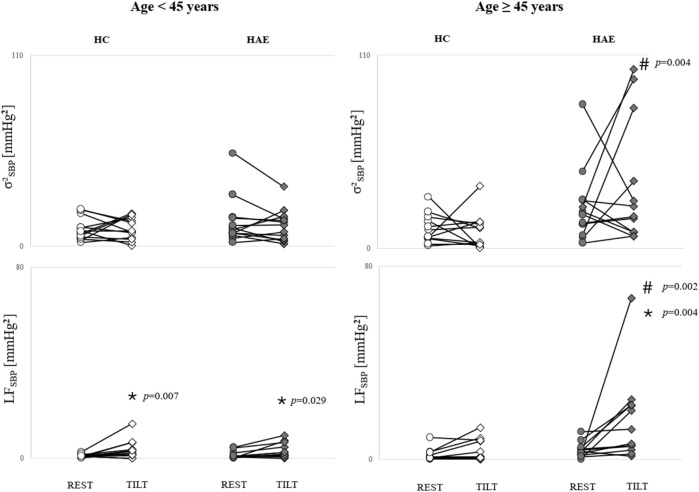
Comparison of the vascular neural control indices in HAE patients and HCs who were younger and older than 45 years during HUTT. Line plots show σ^2^_SBP_ and LF_SBP_ of HAE patients and their age- and sex-matched HCs who were younger (left) and older (right) than 45 years *p < 0.05 REST vs. TILT, #*p* < 0.05 HAE patients vs. HCs, based on two-way analysis of variance with Holm–Sidak *post hoc* test.

The results of the subgroup analysis in patients and HCs (younger and older than 45 years) revealed that all the cardiac control indices were similar in HAE-C1INH patients and HCs, regardless of age, in REST and TILT (data not shown). Regarding vascular control indices, in the younger group, µ_SBP_, σ^2^_SBP_ and LF_SBP_ were similar in HAE-C1INH patients and HCs in both experimental conditions. LF_SBP_ increased from REST to TILT in both HAE-C1INH patients and HCs. The other indices remained unvaried. Regarding the older group, HAE-C1INH patients had higher µ_SBP_ than HCs both during REST [126.5 (122.0–134.0) vs. 118.5 (106.5–121.5) mmHg] and TILT [127.5 (113.0–132.0) vs. 111.5 (103.5–117.0) mmHg] and higher σ^2^_SBP_ [21.2 (9.3–59.2) vs. 7.5 (1.9–14.6) mmHg^2^] and LF_SBP_ [9.4 (4.6–22.4) vs. 0.9 (0.2–7.6) mmHg^2^] during TILT. The increase from REST to TILT of LF_SBP_ was detected only in HAE-C1INH patients [3.0 (1.7–4.5) vs. 9.4 (4.6–22.4) mmHg^2^]. The results are summarized in Figure 3.

## Discussion

The endothelial regulation in HAE-C1INH patients during remission and acute attacks is a pivotal and unsolved point in the comprehension of the disease. The evaluation of any possible factor that could contribute to understanding the underlying mechanism deserves careful attention. This study highlights interesting findings about the role of the ANS in cardiac and vascular regulation that can be summarized as follows: i) HAE-C1INH patients had a physiological response of the cardiac neural control to HUTT, which is similar to that of HCs; ii) HAE-C1INH patients had a more pronounced response of the vascular neural control indices to HUTT than HCs; iii) the LTP might not modify the behavior of the cardiovascular neural control of HAE-C1INH patients; and iv) the abnormal response of the vascular neural control to HUTT in HAE-C1INH patients is more evident with advancing age.

Regarding cardiac neural control, both patients and controls registered the expected decrease in µ_RR_ in the tilting phase as a consequence of the cardiac sympathetic activation and a decrease in HF_RR_, indicating a reduction of the cardiac vagal modulation (Montano et al., 1994; Task Force of the European Society of Cardiology and the North American Society of Pacing and Electrophysiology, 1996; Cooke et al., 1999; Marchi et al., 2016). These results indicate that HAE-C1INH patients have physiological cardiac control in response to the stimulus. Our findings contrast with a previous study by Wu et al. (2017), which described enhanced sympathetic modulation and a blunted response to the orthostatic stimulus. These discrepancies could be explained by the changed scenario in the pharmacological treatment available for prophylaxis in terms of efficacy and adverse events (Beard et al., 2022) that could have significantly modified the clinical status of the patients during the remission periods. More than half of the population enrolled in the previous study (Wu et al., 2017) (63% HAE-C1INH patients) was treated with attenuated androgens. Since estrogens are known to influence autonomic balance (typically enhancing parasympathetic activity), danazol’s anti-estrogenic effect may shift the autonomic tone toward sympathetic dominance (Liu et al., 2003). Among patients on LTP, the vast majority (89%) were treated with lanadelumab. When we analyzed the data on the subgroup of patients on LTP, the above-described findings were reproduced. The limited sample size of the group without LTP prevented a direct comparison between the two patient groups. However, as previously mentioned, our analyses do not allow discrimination of medication and disease effects. Therefore, when considering the differences with previous studies (Wu et al., 2017), we cannot exclude that these differences are due not only to the disease itself but also to the type of medication the patients were taking. The aim of this study was not to presume the long-term efficacy of prophylactic therapy based on the collected data but rather to raise a broader question in the context of current disease management and future prospects. The role of LTP, if any, in endothelial regulation is not the matter of this investigation, but even an indirect contribution of LTP to disease control could play a favorable role in stabilizing endothelial reactivity between attacks at the cardiac level.

In our “androgen-free” population, interesting results were obtained by the vascular control characterization as HAE-C1INH patients had a different behavior than HCs. When the whole population was considered, σ^2^_SBP_ was higher in HAE-C1INH patients than in HCs independently of the experimental condition, suggesting a possible higher vascular sympathetic modulation in patients. Notably, the expected increase in LF_SBP_ during the tilting phase was observed only in the HAE-C1INH patients and not in the HCs. This behavior is clarified when the population was stratified according to age. In the youngest group, the expected increase in LF_SBP_ was observed in both HAE-C1INH patients and HCs, with no differences between the groups, while in the older group, HAE-C1INH patients exhibited higher σ^2^_SBP_ during TILT and a greater increase in LF_SBP_ in response to TILT than HCs. The reduced capability of the cardiovascular system to cope with orthostatic challenges with aging is expected (Veerman et al., 1994; Barnett et al., 1999; Catai et al., 2014; Porta et al., 2014; De Maria et al., 2019) and may explain the lack of a significant increase in LF_SBP_ in older HCs. However, the striking increase in LF_SBP_ in HAE-C1INH patients, together with higher values of σ^2^_SBP_ independent from the experimental conditions, are signs of elevated sympathetic vascular modulation and exaggerated response of the vascular neural control to the HUTT. This could be explained by a selective disruption of vascular control caused by the pathology, which becomes apparent when the duration of exposure to the disease is prolonged. This finding is consistent with observations in many other diseases (Jiang et al., 2022) characterized by a low-grade, chronic inflammation. The intricate relationship between the contact system, inflammatory cytokines, and the ANS makes it challenging to distinguish the causes from the effects. In this study, this point is not directly addressed. On the other hand, the kinetics of the inflammatory markers during an acute stimulus are not aligned with the HUTT protocol, making it difficult to match the plasmatic changes with the orthostatic challenge.

Several limitations of our study should be acknowledged. First, the *post hoc* power analysis for the vascular control indices associated with the primary outcome yielded a value of 70%; hence, our findings require confirmation in future research. However, despite the limited statistical power, our results are highly relevant to those dealing with the complexity of collecting reliable data in a rare disease. Our data update some evidence about the involvement of the ANS in HAE-C1INH patients treated with new therapies and may add insights to design adequately powered future studies.

Second, the BMI difference between the groups was unexpected and of unclear significance with respect to its effects on RR and BP variability. The two groups showed similar ANS profiles at baseline as values in both groups fall within or close to the normal range for Western populations. Previous studies addressing BMI-related effects on RR variability (Altini and Plews, 2021) and BP variability (Chen et al., 2018) were conducted in cohorts under different conditions. Nevertheless, future studies should include BMI as one of the matching variables when selecting the controls, alongside age and sex.

Finally, the threshold of 45 years to distinguish younger and older patients could be considered debatable; however, given that sample size is a critical issue in studies of rare diseases, this choice provided the most pragmatic and statistically balanced approach to evaluate the effect of the disease as age increases.

## Conclusion

Middle-aged HAE-C1INH patients during remission are characterized by elevated vascular sympathetic modulation and preserved cardiac modulation in response to orthostatic challenge. The selective vascular involvement becomes more pronounced with longer disease exposure. The role of currently available long-term prophylaxis in overall disease control, beyond merely controlling acute attacks, will be further clarified in the near future, as sustained optimal disease management becomes achievable for the majority of patients.

## Data Availability

The raw data supporting the conclusions of this article will be made available by the authors, without undue reservation.

## Abbreviations

ANS, autonomic nervous system; BP, arterial blood pressure; BK, bradykinin; ECG, electrocardiogram; HAE-C1INH, hereditary angioedema due to C1-inhibitor deficiency; HCs, healthy controls; HF, high frequency; HF_RR_, power of the RR series in the HF band; HRV, heart rate variability; HUTT, head-up tilt test; IQR, interquartile range; ITACA, Italian Network for Hereditary and Acquired Angioedema; LF, low frequency; LF_SBP_, power of the SBP series in the LF band; LTP, long-term prophylaxis; µ_RR_, mean of the RR series; µ_SBP_, mean of the SBP series; RR, RR interval; SBP, systolic arterial blood pressure; σ^2^_RR_, variance of the RR series; σ^2^_SBP_, variance of the SBP series.
